# *Helicobacter pylori* the Latent Human Pathogen or an Ancestral Commensal Organism

**DOI:** 10.3389/fmicb.2018.00609

**Published:** 2018-04-03

**Authors:** Jackie Li, Guillermo I. Perez-Perez

**Affiliations:** ^1^Department of Medicine, School of Medicine, New York University, New York, NY, United States; ^2^Department of Medicine and Microbiology, School of Medicine, New York University, New York, NY, United States

**Keywords:** *H. pylori*, pathogen, commensal, microbiome, peptic ulcer, gastric cancer

## Abstract

We dedicated this review to discuss *Helicobacter pylori* as one of the latest identified bacterial pathogens in humans and whether its role is mainly as a pathogen or a commensal. Diseases associated with this bacterium were highly prevalent during the 19th century and gradually have declined. Most diseases associated with *H. pylori* occurred in individuals older than 40 years of age. However, acquisition of *H. pylori* occurs mainly in young children inside the family setting. Prevalence and incidence of *H. pylori* has had a dramatic change in the last part of the 20th century and beginning of the 21th century. In developed countries there is a clear interruption of transmission and the lowest prevalence is observed in children younger than 10 years in these countries. A similar decline is observed but not at the same level in developing countries. Here we discuss the impact of the presence or absence of *H. pylori* in the health status of humans. We also discuss whether it is necessary or not to establish *H. pylori* eradication programs on light of the current decline in *H. pylori* prevalence.

## Introduction

Looking back at the history of medical bacteriology, during the last 20 years of the 19th century and for most of the first half of the 20th century, bacteriologists and infectious disease specialists have isolated and identified most of the pathogenic bacteria affecting humans. *Helicobacter pylori* probably represented one of the latest infectious bacteria isolated from humans.

The earliest description of spiral bacteria dates back to 1893 in the stomachs of dogs (Steer, 2002). Thirteen years later the same findings were discovered in human stomachs (Bizzozero, 1893; Krienitz, 1906). Despite the high prevalence of the microorganism demonstrated later, its discovery occurred only at the beginning of the 1980’s. There are several reasons that may explain the delay in identifying and characterizing this prevalent microorganism. Bacteriologists and physicians assumed the high acidity present in stomachs did not provide the ideal condition for microbial colonization, but it turned out the human stomach provided the ideal niche for *H. pylori*. Furthermore, before 1970 gastric biopsies were not routinely obtained for medical analysis and endoscopies were not a common medical practice (Egan and O’Morain, 2007).

The isolation and characterization of *H. pylori* did not occur until 1983 (Marshall, 2002) and this event produced a major multidisciplinary interest including gastroenterologists, microbiologists, and infectious disease specialists among others. After 1983 it was evident that *H. pylori* was colonizing more than half of the world population. As a result of this, *H. pylori* is among the five most studied bacterial organisms in the last 20 years of the 20th century.

To explain this phenomenal global interest in *H. pylori*, we need to remember that as a result of vaccine use, the introduction and globalization use of antibiotics, and the progress and improvement in public health policies, an impressive and steady decline in morbidity and mortality associated with infectious diseases during most of the second half of the 20th century have been documented (Armstrong et al., 1999). At the time of *H. pylori’s* discovery, most of the others infectious diseases, have been controlled or prevented and only few infectious diseases such as tuberculosis, influenza, malaria and HIV remain as major unresolved infectious diseases, mainly in developing countries.

## Historical Perspective

The publication of the presence of *H. pylori* in the gastric human stomach generated radical changes in the medical concepts established before 1980 by gastroenterologists and bacteriologists (Warren and Marshall, 1983). The presence of *H. pylori* in the gastric mucosa was initially associated with an inflammatory process named gastritis, but more relevant from the clinical point of view was the association of this bacterium with peptic ulcer diseases (NIH, 1994). This etiological association was first insinuated by Steer in 1975 and followed by Warren in 1979 and finally confirmed by Warren and Marshall in 1984 (Steer, 2002; Egan and O’Morain, 2007).

Peptic ulcer diseases were very prevalent during the 19th century and the beginning of the 20th century (Susser and Stein, 2002). The only recognized etiologic agent associated with gastric and duodenal ulcers was the production of gastric acid. The conventional concept of “no acid-no ulcer” was very well established in the medical community at the time. After the isolation of *H. pylori* in 1983, it took several years for health professionals to accept the etiologic role of this microorganism in peptic ulcer diseases.

There are two different theories regarding the role of *H. pylori* in peptic ulcer diseases. One theory proposes the role of *H. pylori* as the etiological agent of a modern disease: peptic ulcer disease and as a major risk factor for the development of distal gastric cancer. A series of studies suggested that peptic ulcer disease and gastric cancer are diseases related to modern times. The prevalence and incidence of these diseases rose at the end of the 18th century and peaked by the 19th century. By the beginning of the 20th century there was a steady decline in the number of new and existing cases of peptic ulcer disease and gastric cancer (Susser and Stein, 2002; Sonnenberg et al., 2002). At the same time, the incidence and prevalence of these diseases were not mentioned or associated with a major health human problem before the second half of the 18th century (Sonnenberg and Baron, 2010; Sonnenberg, 2011).

The other theory recognizes that peptic ulcer disease and gastric cancer have been major human health problems since the beginning of human civilization. The evidence to support this idea is based on historic clinical case descriptions that resemble these diseases. The first human peptic ulcer classification dates back to the Western Han Dynasty of a man who died in 167 BCE (Graham, 2014). However, descriptions of this disease were rare and there were no descriptions of gastric cancer.

There is a potential explanation that may account for both conflicting points of view of *H. pylori’s* etiological role in peptic ulcer disease and as the major risk factor for the development of gastric cancer. Peptic ulcer disease and gastric cancer are indeed very ancient diseases, but because the number of cases related to these diseases was insignificant until the second half of the 18th century they have been suggested to be modern diseases. However, increased life expectancy in new industrialized countries, specifically the UK and US, may explain the escalation of peptic ulcer disease and gastric cancer cases. Several studies indicated major changes in life expectancy, from less than 45 years in the 18th century to more than 60 years by the 19th century in the same countries (Colchero et al., 2016) (**Table 1**). This jump in longevity among the human population provides sufficient time for *H. pylori* to express its latent pathogenic capabilities in the development of these major clinical outcomes in colonized individuals. This explanation is in agreement with Graham who suggested that these are not new diseases, but that they have recently become a popular diagnosis caused by increased prevalence due to longer life expectancies and/or change in diagnosis from changing clinical manifestations with time (Graham, 2014). Diagnostic methods for the detection of peptic ulcer disease and gastric cancer were not available prior to the 20th century when medical descriptions of lesions were based exclusively on autopsy.

**Table 1:** Life expectancy according to time and location in the world for human populations^∗^.

Country	Year	Life expectancy^∗∗^	Comment
Liberia	1820–1843	3 years	High mortality
Ukraine	1933	18 years	High mortality
Trinidad	1813–1816	19 years	High mortality
Iceland	1882	20 years	High mortality
Sweden	1751–1859	39–43 years	Longitudinal study
Sweden	1900–1909	58 years	Longitudinal study
Sweden	1925–1934	63 years	Longitudinal study
Sweden	1950–1950	75 years	Longitudinal study
Sweden	2000–2009	84 years	Longitudinal study
England	1600–1725	38 years	Pre-modern era
India	2013	68 years	Developing country
Russia, China, United States, and Japan	2013	75–83 years	Developed countries

*^∗^Colchero et al. (2016); ^∗∗^Defined as the mean age at death.*

## The Role of *H. pylori* as a Pathogen

Despite the multiple studies on *H. pylori*, there are still unanswered questions about the role of this bacterium as a true pathogen or as a commensal organism when colonizing the human stomach.

The role of *H. pylori* as a true pathogen has been the center of major discussions for many years. Most of the well-known human pathogens such as *Vibrio cholera, Streptococcus pyogenes, E. coli* among others are capable of producing an acute infectious disease process with nearly identical symptoms in both pediatric and adult populations. In addition, virulence factors have been identified and exact mechanisms involved in disease progression have been determined. In contrast, the infectious process in *H. pylori* is chronic and only one in ten colonized individuals, most commonly the elderly, develop clinical manifestations years after. Virulence factors have been described in *H. pylori*, but the presence or absences of these factors are not a critical factor in disease development despite that multiple reports indicated that some virulence factors such as CagA are relevant in disease progression and also confirmed using animal models. (Cover and Peek, 2013; Backert et al., 2016; Cover, 2016). This is very important point because we know that most of the world population is colonized with *H. pylori* and that colonization occurs at early age. Furthermore, colonization occurs with *H. pylori* carrying or not carrying those virulence factors, but disease is not expressed until more than 40 years later and the disease only occur in less than 10% of those colonized individuals. This low incidence of disease clearly indicated that *H. pylori* is not a true pathogen and the virulence factors play a small role in disease outcome.

It is widely accepted that colonization with *H. pylori* induces an inflammatory response at the gastric mucosa level (O’Morain, 2006). This natural immune reaction does not produce any symptomatology and it is persistent and variable in intensity. Chronic gastritis represents the latent and potential risk for more severe complications, including peptic ulcer disease, gastric MALT–lymphoma and distal gastric cancer that represent the most relevant clinical manifestations of *H. pylori* pathogenesis (Chey et al., 2017). However, it is important to remember that those severe diseases or complications occur mainly in adults older than 40 years of age and are rare in pediatric populations. Because of this age specific incidence of diseases, it is apparent the colonization with *H. pylori* requires a long time of establishment and the continuous stimulation of the inflammatory response to produce enough histological deterioration for disease expression. Serious complications of *H. pylori* colonization are observed in only 10% of the infected individuals, indicating that *H. pylori* is more of an opportunistic or latent pathogen rather than a true pathogenic bacterium, particularly in adults.

## The Role of *H. pylori* as a Commensal

Because of its low virulence and the fact that disease expression is observed mostly in elderly infected individuals as mentioned above, *H. pylori* could be considered a commensal organism and only an opportunistic pathogen. *H. pylori’s* colonization of the human stomach occurs early in life and transmission is mainly in a family setting. In addition, there is evidence that the association between *H. pylori* and the human host existed for more than 3,000 years based on the detection of this bacterium in the feces from a mummified human (Allison et al., 1999). However, some investigators have suggested that this association is even older. Recent reports of the co-evolution of the gut microbiota in primates (human and non-human) indicate that the colonization of gastric and intestinal bacteria has occurred since the development and evolution of Homo sapiens (Ochman et al., 2010). The sophisticated and specific association of the intestinal microbiota with a specific host clearly indicated a co-evolution between these organisms and their host (Moeller et al., 2016). This new evidence of a specific and unique intestinal microbiota in each primate species support the previous finding of phylogeographic differences that were described in *H. pylori* strains isolated from different regions of the world and from different ethnic groups (Falush et al., 2003; Olbermann et al., 2010).

## Interruption in the Transmission of *H. pylori*

The exact mechanism of transmission of *H. pylori* has not been determined despite almost 35 years of research in this area. However, an interesting epidemiological phenomenon has been occurring, a gradual decrease in the prevalence of *H. pylori* infection and the consequent decline in the incidence and prevalence of peptic ulcer diseases and distal gastric cancer (El-Serag and Sonnenberg, 1998; Sonnenberg, 2011). The decrease in *H. pylori* prevalence is more apparent in developed countries, but is also expected to occur in developing countries.

**FIGURE 1:**
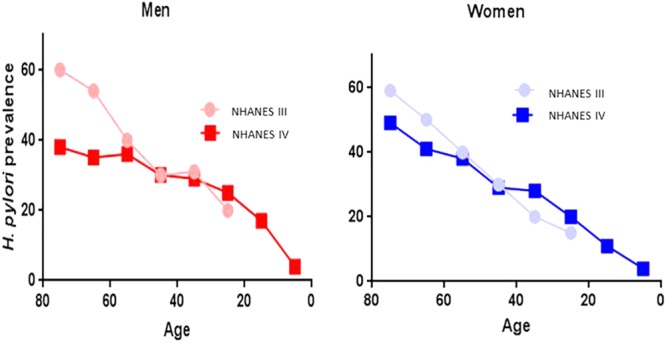
Decline in *H. pylori* prevalence observed in two population studies NHANES III Phase I (1988–1991) (○) and NHANES IV 1999–2000 (□), according to age and gender (blue-women and red-men) in the US (Chen and Blaser, 2008).

**FIGURE 2:**
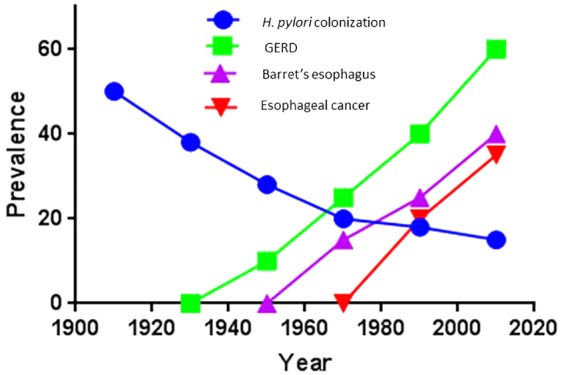
Current trends in esophageal diseases in relation to the gradual decline in *H. pylori* colonization specific for Caucasian populations in developed countries (Hunt and Yaghoobi, 2017).

**FIGURE 3:**
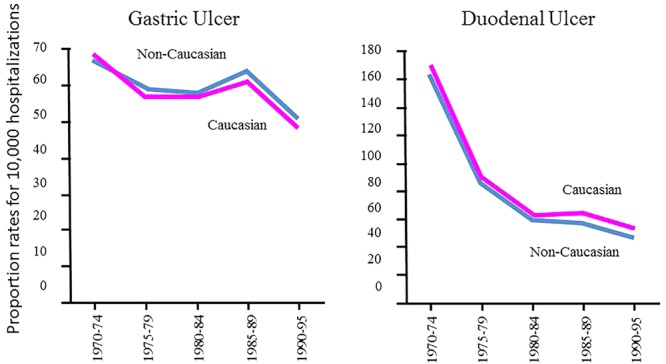
Gradual decline in gastric and duodenal ulcer diseases associated to *H. pylori* infection in Caucasian and non-Caucasian men using the US Department of Veterans Affairs database from 1970 to 1995, prevalence shown as the proportional rate per 10,000 hospitalizations and the Vital Statistics of the United States obtained by the National Center of Health Statistics (NCHS) (El-Serag and Sonnenberg, 1998).

The decline in *H. pylori* prevalence in relation with time was first reported in 1997 (Haruma et al., 1997), and later confirmed in two large population based studies in the United States (Chen and Blaser, 2008) (**Figure 1**) Similar results of the decline of *H. pylori* prevalence have been reported in the Netherlands (den Hollander et al., 2015) and more recently in Japan (Urita et al., 2013). As a result of *H. pylori’s* gradual decline, a series of negative consequences have been described. There has been an alarming increase in asthma (Eder et al., 2006; Holster et al., 2012), a notable increment in body weight that is reaching pandemic proportion of obesity (Blaser, 2012), as well as a potential increase in the susceptibility to diarrheal diseases (Rothenbacher et al., 2000). However, a more serious consequence in the decline of *H. pylori* is the escalation of esophageal diseases such as, GERD, Barrett’s esophagus and adenocarcinoma of the esophagus (Hunt and Yaghoobi, 2017) (**Figure 2**), which have been contributing the surge in Gastroenterology consultations. At the same, time the decline of *H. pylori* prevalence has been directly correlated with a decline in distal gastric cancer and peptic ulcer disease (El-Serag and Sonnenberg, 1998) (**Figure 3**).

**FIGURE 4:**
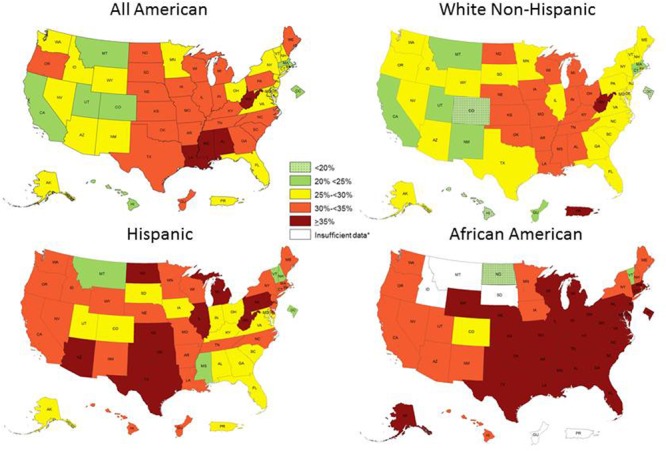
Obesity prevalence according to ethnicity in the US in 2015. Modified from reference 35.

The increasing prevalence of obesity among human populations is an intriguing worldwide health problem that has been occurring in the last 30 years. This problem has become pandemic with no indication of improvement. In particular, the CDC has clearly documented in the US a dramatic increase in obesity in all 50 states from 1990 to 2015 (Centers for Disease Control and Prevention [CDC], 2017) (**Figure 4**). Originally, it was proposed that *H. pylori* prevalence was associated with the increment in obesity. Early reports indicated that successful eradication of *H. pylori* was associated with an increase in body mass index (BMI) (Azuma et al., 2002). Further studies confirmed the role of *H. pylori* in the regulation of hormones related to appetite like ghrelin and leptin Francois et al. (2011). However, the fast increment in obesity rates around the world is not comparable with the speed of *H. pylori’s* decline. The fact that obesity is also affecting populations of low socio-economic level, where *H. pylori* prevalence is still very high, may indicate that the decrease in *H. pylori* infections and the current problem of obesity are not related.

## Is It Really Necessary to Eradicate *H. pylori*?

Based on the steady decline in incidence and prevalence of *H. pylori* in developed countries as well as in developing countries, one of the most interesting questions in the last 5 years is whether or not is necessary to eradicate *H. pylori* from those asymptomatic colonized individuals to prevent future clinical complications. Despite the evidence in *H. pylori* prevalence decline, clinicians, public health personnel and researchers have not reached a consensus. In some countries a national program to eradicate *H. pylori* has been established. The main argument for the establishment of these programs is that existing populations with a high risk to develop severe diseases associated to *H. pylori*, like Japan, that justify its implementation due the high prevalence of gastric cancer (Sugano, 2016). However, before applying this massive eradication policy we need to document the interesting changes in *H. pylori’s* epidemiology and tendencies.

A gradual and consistent decline in the incidence and prevalence of peptic ulcer disease and gastric cancer has been reported in nearly all developed countries (El-Serag and Sonnenberg, 1998; Sonnenberg, 2011) (**Figure 3**). It is expected that a similar decline will take place in developing countries. The most logical and feasible explanation for the decline in the prevalence of these diseases is the gradual and consistent decline in *H. pylori’s* prevalence, which has been occurring several years prior to the discovery of this organism and years prior to the use of antibiotics for its eradication.

**FIGURE 5:**
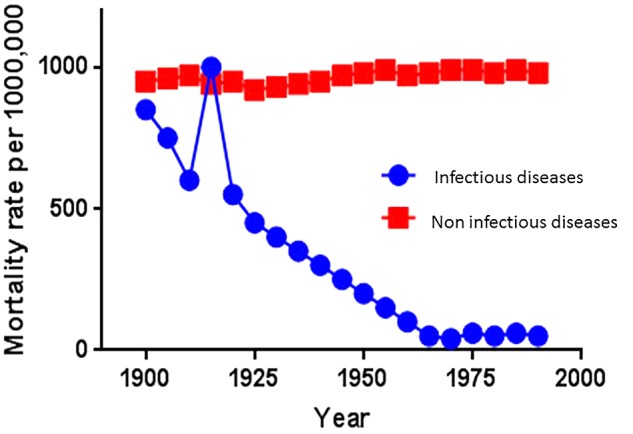
Tendency of human mortality rates associated with infectious diseases and non-infectious diseases in the US during the 20th century (Armstrong et al., 1999).

An important and difficult question to answer is how to explain this steady decline in *H. pylori* prevalence in modern human populations. It is possible that we have not fully understood the transmission of this bacterium. Clearly the improvement of sanitary conditions, availability of clean drink water, changes in family size and many other factors have contributed to block *H. pylori* transmissibility in the same way that all those factors have contributed in the dramatic decline of mortality due to infectious diseases during the 20th century and part of 21th century (Armstrong et al., 1999) (**Figure 5**).

## Conclusion

The sharp and dramatic decline of mortality attributed to infectious diseases during the 20th century is without a doubt one of the major achievements in modern medicine. As a result of the decline of the infectious disease processes, there is now a better opportunity to study chronic human diseases and their potential association with the normal human microbiota.

Most of the systematic studies of the human microbiota were initiated in early 2005, but the role of the human microbiome and its interaction with human immunology and physiology has been known for many years. Its relevance was more clearly define when the hygiene hypothesis was proposed in 1989 (Stiemsma et al., 2015) to explain the sudden increase in certain chronic or immunological diseases that correlated with the notable decline in bacterial, viral and parasitic diseases.

**FIGURE 6:**
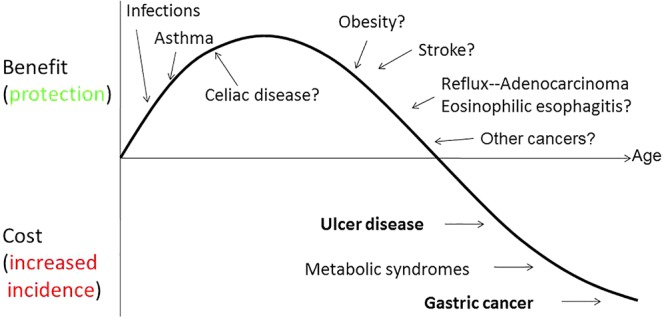
Model of the biphasic nature of *H. pylori* interaction with humans (Atherton and Blaser, 2009).

One of the first associations noticed was between the rapid disappearance of *H. pylori* and the emergence of other diseases. *H. pylori* has been prevalent in human populations, however, as a result of the dramatic decline of prevalence in many developed countries other diseases have emerged. The gradual decline in *H. pylori* prevalence, particularly in Caucasian populations in developed countries has been associated as an attributed risk for the development of gastric esophageal disease (GERD), Barrett’s esophagus, and adenocarcinoma of the esophagus (Chen and Blaser, 2007; Rutsgi and El-Serag, 2014). This potential beneficial role of *H. pylori* infection has also been reported in atopy, allergy, and asthma (Shiotani et al., 2008; Miftahussurur et al., 2017). We do not know if the decline of *H. pylori* infections is the cause of these emerging diseases or if it is just an indicator of the hygiene hypothesis (**Figure 6**).

The increase of all these diseases coincided not only with the decline of *H. pylori’s* prevalence but also with improved socio-economic conditions, use of antibiotics as therapeutic agents, improved quality in drinking water and food. In addition to the decrease of *H. pylori*, humans have witnessed the decline in incidence and prevalence of many of the infectious diseases that were prevalent in the first half of the 20th century (Strachan, 1989).

Finally, one of the major arguments used to support the “test and treat” approach is the high cost of human lives as result of the major clinical consequence of *H. pylori* infection that is distal gastric cancer. We already mentioned that the incidence and prevalence of gastric cancer is gradually decline in both developed and developing countries. Despite this decrease it is estimated that the number of gastric cancer will be increase in the next decades as result of the increase in the expectancy of life in the human populations around the world. However, it is possible to treat only those individuals with high risk to develop gastric cancer or establish programs for early detection of gastric cancer without perform massive eradication programs of this bacterium that may be important to colonize the gastric stomach of young human beings.

## Author Contributions

Both of the authors were involved in the conception, writing, and discussion of this review.

## Conflict of Interest Statement

The authors declare that the research was conducted in the absence of any commercial or financial relationships that could be construed as a potential conflict of interest.
